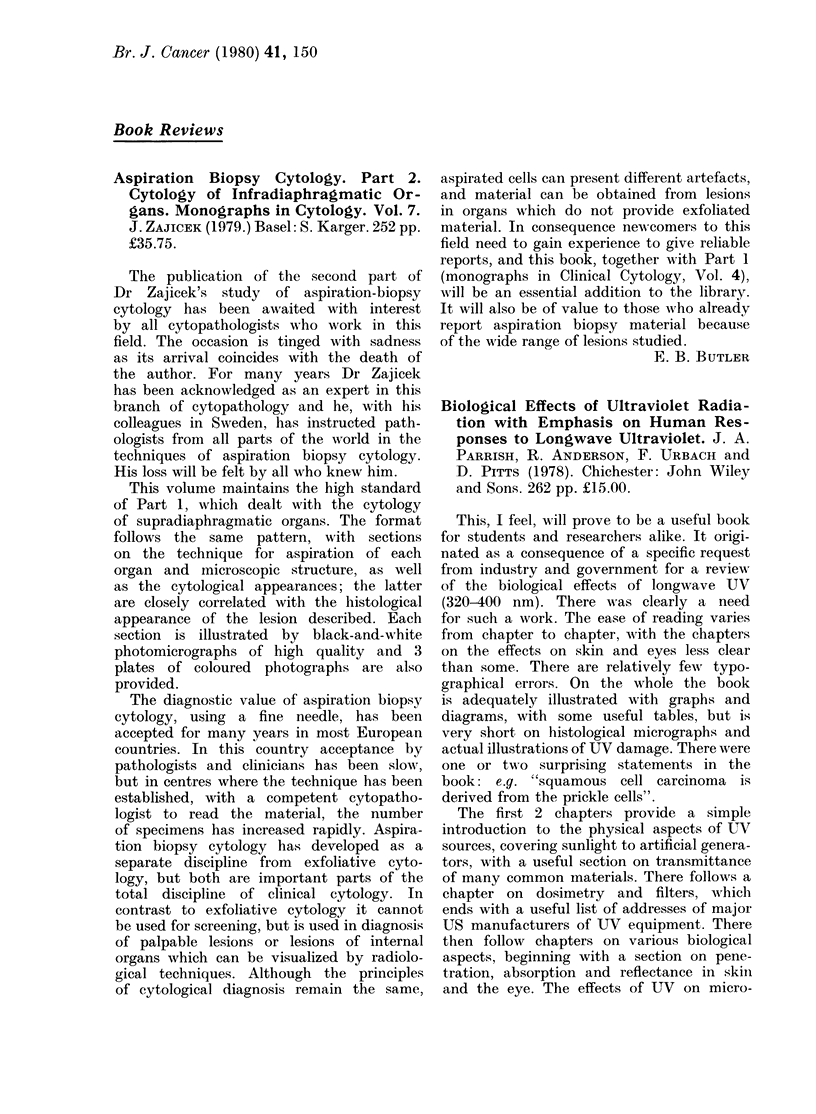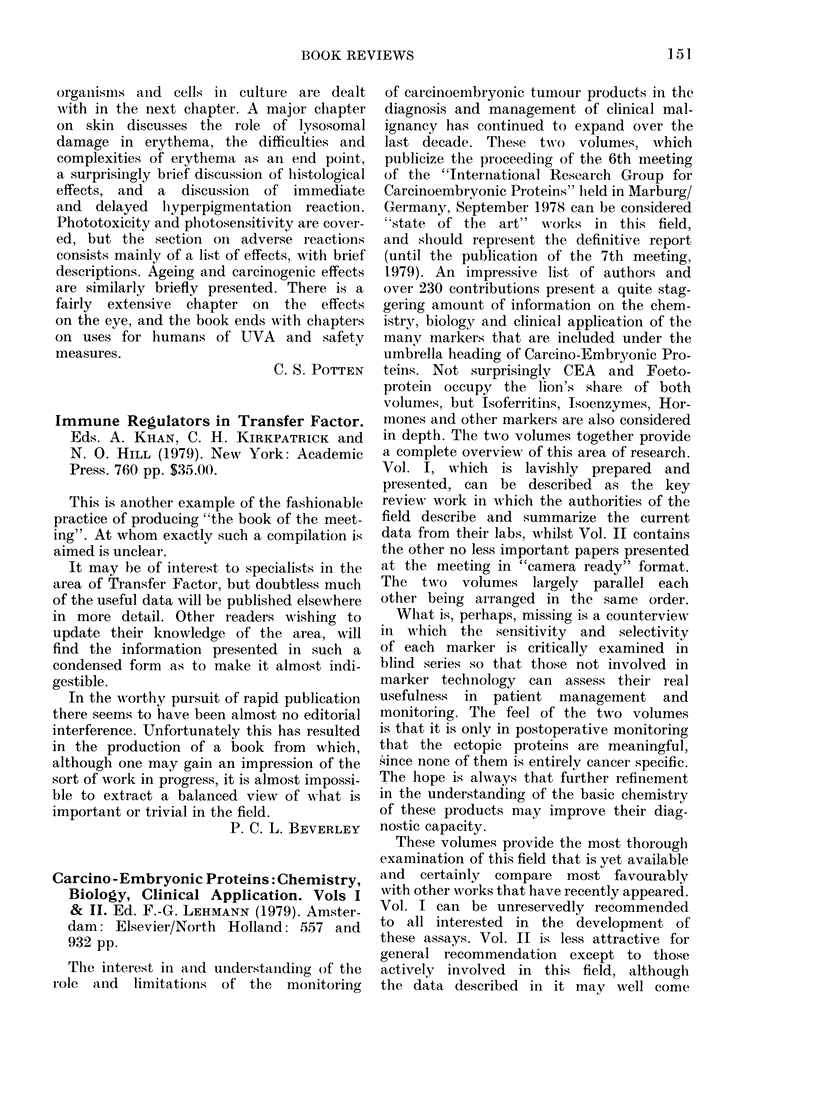# Biological Effects of Ultraviolet Radiation with Emphasis on Human Responses to Longwave Ultraviolet

**Published:** 1980-01

**Authors:** C. S. Potten


**Biological Effects of Ultraviolet Radiation with Emphasis on Human Responses to Longwave Ultraviolet.** J. A. Parrish, R. Anderson, F. Urbach and D. Pitts (1978). Chichester: John Wiley and Sons. 262 pp. £15.00.

This, I feel, will prove to be a useful book for students and researchers alike. It originated as a consequence of a specific request from industry and government for a review of the biological effects of longwave UV (320–400 nm). There was clearly a need for such a work. The ease of reading varies from chapter to chapter, with the chapters on the effects on skin and eyes less clear than some. There are relatively few typographical errors. On the whole the book is adequately illustrated with graphs and diagrams, with some useful tables, but is very short on histological micrographs and actual illustrations of UV damage. There were one or two surprising statements in the book: *e.g.* "squamous cell carcinoma is derived from the prickle cells".

The first 2 chapters provide a simple introduction to the physical aspects of UV sources, covering sunlight to artificial generators, with a useful section on transmittance of many common materials. There follows a chapter on dosimetry and filters, which ends with a useful list of addresses of major US manufacturers of UV equipment. There then follow chapters on various biological aspects, beginning with a section on penetration, absorption and reflectance in skin and the eye. The effects of UV on microorganisms and cells in culture are dealt with in the next chapter. A major chapter on skin discusses the role of lysosomal damage in erythema, the difficulties and complexities of erythema as an end point, a surprisingly brief discussion of histological effects, and a discussion of immediate and delayed hyperpigmentation reaction. Phototoxicity and photosensitivity are covered, but the section on adverse reactions consists mainly of a list of effects, with brief descriptions. Ageing and carcinogenic effects are similarly briefly presented. There is a fairly extensive chapter on the effects on the eye, and the book ends with chapters on uses for humans of UVA and safety measures.

C. S. POTTEN